# The Institutional Worker

**Published:** 1912-08-24

**Authors:** 


THE
### INSTITUTIONAL WORKER
**Being a Special Supplement to "The Hospital"**

### Editor's Notices.

**Contributions:**

Contributions should be written, or preferably typed, on one side of the paper only, and all articles sent in are accepted upon the distinct understanding that they are forwarded to The Hospital only.

The Editor cannot undertake to return MSS. not used, but when a stamped directed envelope is enclosed the MSS. may be returned if a special request is made.

Accepted articles and paragraphs of news will be paid for after publication at the scale rate.

**Address:**

To prevent delay all contributions and letters on editorial business must be addressed exclusively to the Editor, "The Hospital," 29 Southampton Street, Strand, London, W.C. It is important that this regulation shall be strictly observed.

**Correspondence:**

Correspondence on all subjects is invited. The name and address of correspondents must be given as a guarantee of good faith, but not necessarily for publication.

**Special Articles:**

Special articles are invited, and questions, inquiries, and paragraphs upon all matters relating to the work, administration, and management of general, special, mental, fever, cottage and convalescent hospitals, sanatoria, homes, institutions, societies and organisations for the treatment or care of the sick, injured and dependants of all classes. Every matter affecting the interests and welfare of the staff of all grades working in these institutions will receive special consideration.

**Administrative Medicine, Research and Items of News:**

Special terms will be paid for approved articles on subjects in this wide field by experts who have made some section of it their special study and interest. We wish to give prominence to every movement tending to advance accuracy of diagnosis, efficiency in treatment, and the development of modern methods to eradicate disease and promote the welfare of the suffering.

**A Bureau of Information:**

We invite inquiries and applications for information and help in providing for that numerous class of sufferers who require special care or house-room which they cannot obtain in their own homes or secure for themselves. **We cannot, however, prescribe, or recommend practitioners.**

**Books for Review:**

Publishers are particularly requested to send advance proofs of any new books of importance whenever possible, as the Editor has made arrangements to publish immediate reviews on a new plan.

**Photographs, Plans, Blocks, and Illustrations:**

It is requested that wherever possible MSS. may be accompanied by illustrations in any of the above forms. The name of the sender and of the article to which it belongs should be written on the back of each photograph, drawing, or block for purposes of identification.

**Local Papers:**

Newspapers containing reports or news paragraphs **should be marked** and addressed to the Sub-Editor.

**The Coupon System:**

A coupon will be found at the bottom of the third inside page of the cover each week. This coupon must be attached to every question or inquiry to which an answer is desired in The Hospital.

### An Important Point.

So rapidly increasing is the circulation of The Hospital that it is often a matter of considerable difficulty to obtain a copy of the Journal at the local newsagents, especially so in some of the smaller Provincial towns. In these circumstances we would call the attention of our readers to the distinct advantages to be gained by *Subscribing* to The Hospital either through a Bookseller or direct to the Publishing Offices. By such means you are sure to obtain the Journal almost immediately on publication; it reaches you regularly on a certain day and at a fixed hour; you know when you may expect it, and there is no fear of being told that it is sold out. This is a great help when seeking a post, as it enables you to send in your application without delay, an advantage which may secure you the appointment. In addition to this, as a Subscriber, no matter how many times you may change your address, or in what part of the country you may be at the moment, a postcard will ensure that you receive The Hospital on the same day and at the same hour as when at home.

**Subscriptions,** which may commence at any time, are payable in advance. Cheques and Post-Office Orders should be crossed London County and Westminster Bank, Covent Garden Branch, and made payable to the Manager of The Hospital.

Rates of Subscription (including Postage).

| *United Kingdom.* | | *Foreign and Colonial.* | |
|---|---|---|---|
| Three Months ... | ... 2s. 0d. | Three Months ... | ... 2s. 6d. |
| Six Months ... | ... 4s. 0d. | Six Months ... | ... 5s. 0d. |
| Twelve Months ... | ... 6s. 6d. | Twelve Months ... | ... 8s. 8d. |

*SUBSCRIPTION FORM.*

*Please send me* The Hospital *for..................months, commencing with your issue of.............................., for which please find enclosed.............................*

*Name*...................................................

*Address* ..........................................................

..........................................................

*Date*......................................

*To* .................................... *Newsagent.*

### Manager's Notices.

**All letters on business matters must be addressed to The Manager, "The Hospital," 28 and 29 Southampton Street, London, W.C.**

**Advertisements:**

To ensure insertion, all advertisements for The Hospital must reach the Manager not later than Wednesday at noon in each week. For scale of charges see pages v and 2.

**Remittances:**

**It is especially requested that remittances be made by Cheque or Postal Order, and in halfpenny stamps only when the amount is under sixpence.**

### Books for Institutional Workers.

"Midwife's Pocket Dictionary, with revised Rules of the C.M.B." ... 1s. 1d.
"The Nurses' Pronouncing Dictionary" ... 2s. 0d
"A Handbook for Nurses." (J. K. Watson, M.D.) 5s. 4d
"Art of Feeding the Invalid." ... 1s. 6d
"Surgical Ward Work and Nursing." ... 5s. 5d
"Massage Movements, including the Nauheim Exercises" ... 1s. 1d.
"Surgical Instruments and Appliances used in Operations" (Harold Burrows, M.B.London, B.S., F.R.C.S.) ... 1s. 8d.
"The Nurse's Materia Medica" (Herbert French, M.D.) ... 2s. 9d.

Case-papers, Charts, Professional Cards, and every kind of Institutional Literature.

Of all Booksellers or of The Scientific Press, Limited. 28 and 29 Southampton Street, Strand, London, W.C.

